# A New Wave of Industrialization of PHA Biopolyesters

**DOI:** 10.3390/bioengineering9020074

**Published:** 2022-02-15

**Authors:** Martin Koller, Anindya Mukherjee

**Affiliations:** 1 Office of Research Management and Service, c/o Institute of Chemistry, University of Graz, NAWI Graz, Heinrichstrasse 28/IV, 8010 Graz, Austria; 2 ARENA—Association for Resource Efficient and Sustainable Technologies, Inffeldgasse 21b, 8010 Graz, Austria; 3 Global Organization for PHA (GO!PHA), Oudebrugsteeg 9, 1012 JN Amsterdam, The Netherlands; anindya.mukherjee@gopha.org; 4 PHAXTEC, Inc., Wake Forest, NC 27587, USA

**Keywords:** biopolymers, commercialization, copolyester, homopolyester, polyhydroxyalkanoate

## Abstract

The ever-increasing use of plastics, their fossil origin, and especially their persistence in nature have started a wave of new innovations in materials that are renewable, offer the functionalities of plastics, and are biodegradable. One such class of biopolymers, polyhydroxyalkanoates (PHAs), are biosynthesized by numerous microorganisms through the conversion of carbon-rich renewable resources. PHA homo- and heteropolyesters are intracellular products of secondary microbial metabolism. When isolated from microbial biomass, PHA biopolymers mimic the functionalities of many of the top-selling plastics of petrochemical origin, but biodegrade in soil, freshwater, and marine environments, and are both industrial- and home-compostable. Only a handful of PHA biopolymers have been studied in-depth, and five of these reliably match the desired material properties of established fossil plastics. Realizing the positive attributes of PHA biopolymers, several established chemical companies and numerous start-ups, brand owners, and converters have begun to produce and use PHA in a variety of industrial and consumer applications, in what can be described as the emergence of the “PHA industry”. While this positive industrial and commercial relevance of PHA can hardly be described as the first wave in its commercial development, it is nonetheless a very serious one with over 25 companies and start-ups and 30+ brand owners announcing partnerships in PHA production and use. The combined product portfolio of the producing companies is restricted to five types of PHA, namely poly(3-hydroxybutyrate), poly(4-hydroxybutyrate), poly(3-hydroxybutyrate-*co*-3-hydroxyvalerate), poly(3-hydroxybutyrate-*co*-4-hydroxybutyrate), and poly(3-hydroxybutyrate-*co*-3-hydroxyhexanoate), even though PHAs as a class of polymers offer the potential to generate almost limitless combinations of polymers beneficial to humankind. To date, by varying the co-monomer type and content in these PHA biopolymers, their properties emulate those of the seven top-selling fossil plastics, representing 230 million t of annual plastics production. Capacity expansions of 1.5 million t over the next 5 years have been announced. Policymakers worldwide have taken notice and are encouraging industry to adopt biodegradable and compostable material solutions. This wave of commercialization of PHAs in single-use and in durable applications holds the potential to make the decisive quantum leap in reducing plastic pollution, the depletion of fossil resources, and the emission of greenhouse gases and thus fighting climate change. This review presents setbacks and success stories of the past 40 years and the current commercialization wave of PHA biopolymers, their properties, and their fields of application.

## 1. Introduction

Plastics based on fossil resources have proven their valuable role in increasing our quality of life in various sectors, as shown by their wide-spread application as packaging materials for food and other perishable goods; in the medical and pharmaceutical field; and in the transportation sector, e.g., in automobiles or aircraft, where plastics have enabled novel technological and safety-related improvements. Thus, it is undisputed that plastics, which ubiquitously accompany us in our daily lives, have made our society more convenient. However, the persistence of fossil plastics at their end of life, the insufficiency of collection and recycling systems, and their leakage into terrestrial and aquatic environments, ultimately leading to microplastic pollution of the eco- and biosphere, are omnipresent threats to all life on earth [1]. A UN study in 2005 concluded that plastic waste in oceans would result in the formation of microplastics and that these microplastics constituted the next environmental threat so great that it had the potential to be the next epic threat [2]. These and the topic of greenhouse gas emissions connected to the production and incineration of fossil plastics have raised significant awareness among consumers and finally among policymakers. While regulations are being put in place to reduce the use of plastics, not all of these measures are necessarily beneficial to the environment and practical from a convenience standpoint. For a real cure to the plastic pollution predicament, real, sustainable solutions are needed [1]. It is important that such solutions forgo the negative environmental and logistical impacts of plastics while retaining their benefits. Alternatives that fulfill all of these criteria have been provided by nature, and such materials already exist. Polyhydroxyalkanoate (PHA) biopolyesters, produced by and playing multifaceted metabolic roles in numerous bacteria and archaea, are expedient examples of materials that bridge the desired benefits of plastics without endangering the environment. For illustration, a recent study by Dilkes-Hoffman et al. reports the complete degradation of PHA bottles in marine environments within 1.5 to 3.5 years, in contrast to the decades or even centuries that disintegration of petro-plastic bottles would take [3].

Similar to other biopolymers such as carbohydrates, nucleic acids, and proteins, PHA biopolymers have been established as macromolecules embedded into the closed cycles of producing and degrading materials in nature: PHAs are produced by living organisms (“biosynthesized” materials) and they biodegrade. Moreover, PHAs are produced from renewable raw materials, thus originating from natural substrates instead of fossil resources. Importantly, PHAs are biocompatible to humans and other life forms and are readily metabolized to non-toxic compounds when ingested by living organisms [4].

The ecological concerns of fossil plastics, together with ongoing limitations of fossil resources, have now opened the door for PHA biopolymers to play a front-running role for industry and society while maintaining nature’s cycle of circularity and sustainability. The skyrocketing crude oil prices in the 1970s prompted the first commercialization efforts in PHAs. However, much of that effort slowed down after the recovery of crude oil prices, although the scientific research continued. Price and availability were identified as the primary obstacles to the continued development and commercialization of PHAs. They were identified as not being price competitive to well-established fossil plastics, and certain challenges in their processability also had to be overcome. The material properties of PHAs do not exactly match those of the fossil-based competitors; in other words, they were not drop-ins for fossil plastics, although they cover a substantial spectrum of their property profile [5]. Now, many decades later, after having accumulated significant knowledge on production, processability, and end-of-life outcomes of PHA, we finally are on the threshold of serious and sustained commercialization efforts of these biopolymers. It is now better understood how the production price can be lowered by resorting to inexpensive or even near zero-cost carbon sources [6,7], which natural microbes are best suited to produce the various types of PHA from a given substrate [8], how microorganisms can be tailored using systems biology and metabolic engineering approaches [9,10], how bioprocesses can be rendered to consume less energy by running PHA production under low-sterility or nonsterile conditions with extremophilic microorganisms [11], and how to optimize downstream processing for recovery of intracellular PHA [12]. In addition, a large body of knowledge has been generated on fine-tuning the (co)polymer composition and thus tailoring the product properties during the bioprocess [13] and on facilitating PHA processing by blending appropriate chemical additives and other polymers [14]. Indeed, a growing number of companies spread over different global regions have started commercial-scale PHA biopolymer production for processing towards vendible items by melt extrusion, injection molding, 3D printing, electrospinning, etc. [15,16]. This new wave of PHA commercialization is increasingly becoming an integral part of current concepts of the bioeconomy and circular economy, which, as postulated by the European “Green Deal” [17], are characterized by the replacement of “end-of-pipe products” such as fossil plastics, especially for single-use applications, by biodegradable alternatives based on renewable carbon to drastically reduce plastic pollution and greenhouse gas emissions and to curb global warming [1]. In fact, calculations based on a plethora of life cycle studies estimate that replacement of 1 kg fossil plastic by PHA could salvage on average CO_2_ emissions by 2 kg and around 30 MJ of fossil resources on an energy basis [18].

**Figure 1:**
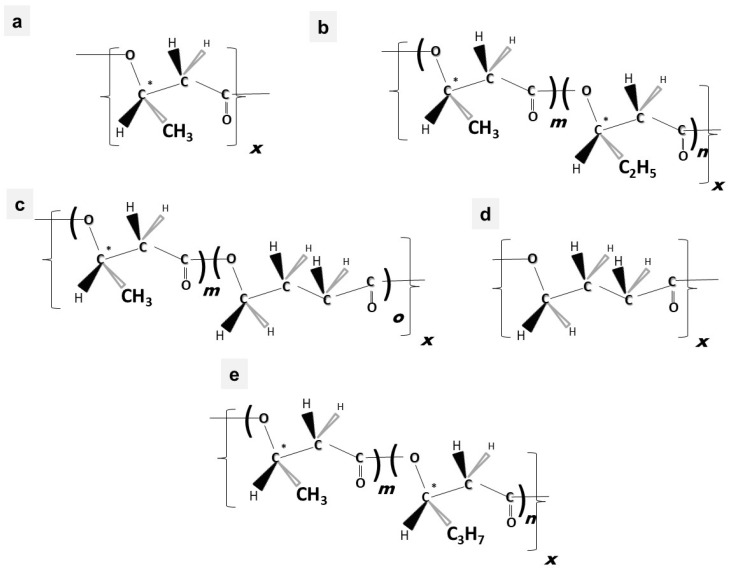
Chemical structures of “bulk PHA” types: (**a**) P(3HB); (**b**) P(3HB-*co*-3HV); (**c**) P(3HB-*co*-4HB); (**d**) P(4HB); (**e**) P(3HB-*co*-3HHx). Asterisks (*) indicate chiral centers of PHA building blocks.

Material properties of PHA biopolymers are dependent on the type and distribution of various monomeric building blocks; PHAs are a versatile group of biomaterials, with characteristics that range from elastomeric to semicrystalline thermoplastic-like polymers [19]. Despite the discovery of more than 150 different hydroxyalkanoate (HA) building blocks that constitute the PHA biopolyester family, only a limited number of PHA copolymer types have reached industrial maturity. As shown in the subsequent sections, we currently witness considerable activities in different regions globally towards commercialization of a few PHA biopolymers, namely the homopolyester poly(3-hydroxybutyrate) (P(3HB)); the copolyesters poly(3-hydroxybutyrate-*co*-3-hydroxyvalerate) (P(3HB-*co*-3HV)), poly(3-hydroxybutyrate-*co*-4-hydroxybutyrate) (P(3HB-*co*-4HB)), and poly(3-hydroxybutyrate-*co*-3-hydroxyhexanoate) (P(3HB-*co*-3HHx)); and, to a minor extent, the homopolyester poly(4-hydroxybutyrate) (P(4HB)) and some medium-chain-length PHA (*mcl*-PHA) copolyesters. The chemical structures of these biopolyesters are illustrated in Figure 1.

While it is challenging to estimate the current volume of PHA produced industrially, it has, to the best of our knowledge, not yet exceeded 10,000 t annually at the time of submission of the present article (January 2022); however, capacity expansions of over 1.5 million t have already been announced for the next 5–10 years, and an additional 1 million t are in the planning stages. Compared with the estimated global plastic production of roughly 400 Mt per year, the share of PHA is negligible [20]. Therefore, this review is dedicated to drawing a clear current and developing picture of a very old biopolymer platform that is transforming into the new “PHA industry”. The intent here is to take the reader on a journey through the history of PHA commercialization attempts, highlighting the obstacles and stumbling blocks that often made this path difficult and the targeted applications where PHA is intended to be commercialized.

## 2. Industrializing Poly(3-hydroxybutyrate) (P(3HB) or PHB)

### 2.1. Challenges of Processing and Commercializing P(3HB)

P(3HB) constitutes the by far best-studied representative of the PHA family. It is also the type of PHA biopolyester that is produced by the largest number of microbes in nature from simple feedstocks such as carbohydrates, alcohols such as glycerol, or fatty acids with an even number of carbon atoms. In addition, many more bacteria convert gaseous C_1_-substrates like methane (by type-II methanotrophs), CO_2_ (by cyanobacteria and aerobic chemolithoautotrophic hydrogen-oxidizing “knallgas bacteria” such as *Alcaligenes eutrophus*—today *Cupriavidus necator*), and syngas (generated from organic waste materials, e.g., by Rhodospirilli) into P(3HB) homopolyester (reviewed by Koller et al., 2017) [21].

PHB, however, has a very narrow melt processing window since its melting temperature is close to its degradation temperature. It is also highly crystalline; therefore, it has a low elongation at break (ε) and is brittle. P(3HB) suffers from slow crystallization rates and lower biodegradability rates compared to other types of PHA, which have been successfully industrially manufactured since the 1980s. However, despite the apparent property deficiencies, P(3HB) also has some beneficial features, especially for the production of hard, creep-resistant items, which do not change their properties over a broad temperature range even when stored for several years. Another beneficial aspect of P(3HB) is the adaptability of its melt viscosity for different processing techniques (reviewed by [5]). According to Urs Hänggi [22], P(3HB) outperforms many competing petrochemical plastics in UV resistance and mechanical stability.

Commercial P(3HB) is also used to blend with other biobased polymers, such as poly(lactic acid) (PLA) and thermoplastic starch (TPS), and synthetic but still biodegradable polyesters such as poly(butylene adipate terephthalate) (PBAT); resulting polymer blends achieved certifications in biodegradability (90% of carbon metabolized within 180 days under standardized conditions according to European norm EN 13432) and compostability (not more than 10% of the polymer remains in a sieve of 2 mm pore size after 180-day composting tests under standardized conditions according to EN 13432) [23]. Incorporation of additives (diverse nucleating agents to increase crystallization rates; stabilizing agents (antioxidants), and plasticizing agents such as tributyl citrate, glycerol, and sorbitol) is often used to overcome the above-discussed shortcomings of the material properties of P(3HB). Moreover, P(3HB) can be processed with filler materials such as lignocelluloses to fine-tune the properties of P(3HB), such as lowering crystallinity and density, increasing biodegradability further, and reducing the production price of the final biobased and biodegradable polymer products. Gregorova et al. [24] have demonstrated many of these improved attributes by incorporating untreated and modified (alkali, stearic acid, or hydrothermal treatment to improve the interface adhesion) beechwood flour in P(3HB). They produced films of P(3HB) blended with lignocellulose by hot compression molding which displayed significant reduction in the degree of crystallinity (66% to about 50% for the composites), while showing improved Young´s moduli, thus demonstrating the reinforcement effect of these fillers [24].

### 2.2. Biomer

Biomer, based in Schwalbach, Germany, was one of the first industrial producers of P(3HB) using the strain *Azohydromonas australica* (formerly known as *Alcaligenes latus*) from sucrose as the carbon feedstock. The Biomer process is based on the experimental work carried out and patented (1983 German Patent No. 379,613 and 1988 Canadian Patent No. 1,236,415) by Lafferty and Braunegg in the 1980s in Graz, Austria. These researchers discovered that *A. australica* is an outstandingly fast-growing bacterium, which accumulates PHA also during balanced growth, in parallel to the formation of catalytically active biomass [5]. The technology based on these experiments was developed by Chemie Linz/PCD Polymere GmbH in Austria in the late 1980s and was later scaled up to an annual production of 2 t P(3HB) in 1991. However, the low crude oil price at the time made PHA less competitive with fossil plastics, discouraging growth in the industrial proliferation of these biopolyesters (reviewed by da Cruz Pradella [20]).

Biomer acquired the base technology and commercialized P(3HB) in 1993. The technology includes several grades of these resins blended with low-molecular-weight softeners and nucleating agents (tributyl citrate). The products are sold to plastics processing companies. Biomer also markets pure P(3HB) in powder form for blending into other biobased polymers such as PLA. Commercial grades include Biomer P209/P209E, P226/P226E, P263/P263E, and P309. “E” refers to the presence of “polymeric nucleating agent” to overcome low crystallization rate, while the others contain boron nitride as a nucleating agent. Their degree of crystallinity (*X_c_*) values range between 60 and 70%, melting temperatures (*T_m_*) range between 170 and 175 °C, glass transition temperature (*T_g_*) ranges between −5 and 5 °C, and weight average molecular weight (*M_w_*) is 500–600 kDa. Tensile strength (σ) ranges between 8 and 27 MPa and elongation at break (ε) between 3.7 and 16%. The property variations are due to the additives such as nucleating agents and plasticizers added. The data show that these P(3HB) homopolyesters are rigid materials suitable for injection or compression molding or melt extrusion and are not sufficiently flexible for film blowing [25]. According to Biomer, their production capacity is 900 t *per annum*; these products were used for many studies developing new materials for different applications (personal communication U. Hänggi [26]). In this context, Arrieta et al. developed composites of PLA and Biomer P226, with the natural terpene limonene acting as a plasticizer, improving the processing, interaction of the polymers, and disintegration of the blend during composting. Transparent films were generated, in which P(3HB) acted as a reinforcement of the PLA matrix, resulting in a higher oxygen barrier and improved surface water resistance, which makes these materials interesting candidates for food packaging purposes. Composting studies showed that P(3HB) rather slowed down the disintegration rate of PLA, while limonene favored the composting process [27]. Recently, Biomer P304 was blended with abundantly available agave fibers and organic peroxide (to improve compatibility between P(3HB) and fibers) and processed via reactive extrusion; the flexural and impact strengths increased compared to the neat P(3HB) by 46% and 45%, respectively. Authors suggest also this material for food packaging applications [28].

### 2.3. PHB Industrial S.A.

Another important example of companies commercializing P(3HB) homopolyester is PHB Industrial S.A. in Brazil (PHB/ISA). Their product BIOCYCLE^TM^ is produced on an annual scale of approximately 50–100 t. In this process, *C. necator* is cultivated in fed-batch mode on hydrolyzed sucrose (equimolar mixture of glucose and fructose); this process is integrated into an energetically autarkic bioethanol and cane sugar factory [29]. This approach is based on technologies originally developed by the Copersucar Technology Center (CTC) at the Institute of Technological Research of São Paulo State (IPT), São Paulo State University, based on a research grant from 1991 for “Production of Biodegradable Plastics from Sugar via Biotechnological Route” and sold in 1995 to PHB/ISA. The PHA production process is integrated into the sugar and bioethanol factory Usina da Pedra, Serrana. Remarkably, the PHB/ISA process is energetically autarkic: lignocellulosic sugar cane bagasse “waste” remaining after sugar extraction is used to fuel the entire industrial plant energetically—not only the bioreactor and medium sterilization and the cultivation process itself for PHA production, but also the bioethanol distillation and water evaporation for sugar crystallization. Moreover, fusel alcohols (amyl alcohol, etc.) are used for extraction of the BIOCYCLE^TM^ PHA (trademark established in 2000) from biomass [20]. P(3HB) homopolyester is sold as “BIOCYCLE 1000” [30]. According to the company, the process proved to be economically feasible in 2004 at a volumetric productivity of about 1.7 g/(L·h) and attaining PHA concentrations of 70 g/L in a 13 m^3^ production bioreactor [31].

### 2.4. Tianan Biologic Materials Co.

In Ningbo, Zhejiang province, People’s Republic of China (PR China), Tianan Biologic Materials Co., a company normally focusing on P(3HB-*co*-3HV) copolyester production [32], also produces and markets P(3HB) homopolyester designated ENMAT Y3000^TM^ (powder form) and ENMAT Y3000P^TM^ (pellet form) for injection molding, thermoforming, and extrusion applications. Tianan produces this “100% biobased and 100% biodegradable” (manufacturer information) product using cassava starch as the raw material, which is sold in the form of slightly brown polymer pellets, containing an undisclosed nucleating agent to induce crystallinity. This product is certified as compostable by the US Biodegradable Products Institute (BPI), is listed as Food Contact Material (FCM) substance No. 744 in Table 1 of Annex I of the Plastics Regulation of the European Union, and has been EU REACH compliant since 2008 [33].

### 2.5. Nafigate Corporation–Hydal

An intriguing approach for P(3HB) manufacturing was developed in Brno, Czech Republic, by the Hydal consortium [34]. Using this technology, Nafigate produces P(3HB) homopolyesters from waste frying oil and resorts to a polymer recovery process using oils, which, as the company claims, makes the entire process about 50% less energy-intensive compared to poly(ethylene) (PE) production. This process achieved a technological readiness level (TRL) of 9 in 2019 and is claimed by Nafigate as “*the first in the World to use 100% waste on an industrial scale… for the production of natural PHA biopolymer*”. Undisclosed additives for stabilization are added to the biopolymer, which is sold as granules in the Czech Republic. Some recommended applications of their polymers are mulch films and 3D printing filaments. In addition, the company uses its own P(3HB) as primary (micro)plastic beads for peeling in shower milk products as a replacement for fossil plastic beads which are banned as primary microplastics in Europe as part of the Intentionally Added Microplastics Legislation. In addition, Nafigate is producing formulations using P(3HB) and substitutes polluting chemicals such as oxybenzone chemical UV filters in sunscreen creams; these products are already available on the market (“Naturetics^TM^” products; market launch in Czech Republic in 2021) [35]. Moreover, the application of the product in biomedicine, sustainable packaging, and smart fertilizers is envisioned. As a timeline, the company expects to start P(3HB) production for biomedical applications in 2022 and to start large-scale P(3HB) production for packaging purposes in 2026. Unfortunately, the company does not disclose production volumes or the downstream processing technique applied; according to the internet site, they do not resort to solvent extraction (“*process involves the breakdown of microbial cells from which PHA granules are released*”). The bioprocess is carried out using wild-type *C. necator* as production strain [34].

### 2.6. Newlight Technologies LLC

In the USA, the Californian company Newlight Technologies LLC, located in Huntington Beach, produces P(3HB) homopolyester by using “*naturally occurring microorganisms found in the ocean*”. Unfortunately, not much is disclosed by the company about its biotechnological process. Bioreactor design, biocatalyst design, purification, and material performance were optimized over a period of ten years from 2007 to 2017. Since 2019, the commercial production plant “Eagle 3” is in operation. Renewable energy is used for this process. According to the company, marine microbes (“Newlight´s 9X biocatalyst”) consume air and CO_2_ from greenhouse gas to accumulate P(3HB) homopolyester, marketed under the trade name AirCarbon^TM^. The products are FDA approved for food contact according to the norm FCN 1754 and “carbon-negative” certified according to the International Organization for Standardization (ISO 14046-3) and the specification for the assessment of the life cycle greenhouse gas emissions of goods and services (PAS 2050: 2008/2011). They are “ocean degradable” according to the American Society for Testing and Material (ASTM D6691 and D7081) and “industrially compostable” according to ASTM D6400 [36]. AirCarbon^TM^-based P(3HB) is used as the base resin for the company´s Restore^TM^ (foodware) and Covalent^TM^ (fashion products) branded products. Newlight sells biodegradable food-contact items such as straws and cutlery that are blue-stained, probably to look like the ocean. The company also claims these products to be durable and dishwasher safe [37]. Products branded Covalent use AirCarbon^TM^ P(3HB) for the production of fashionable eyewear, and even as a leather replacement in smartphone covers and other accessories. These products have been being marketed since the end of 2020. Interestingly, Covalent^TM^ puts a “carbon date”—a unique timestamp placed on every Covalent^TM^ product to disclose the time when the AirCarbon^TM^ P(3HB) used to manufacture the product was biosynthesized. When entering this “carbon date” into the company´s website, the consumer can access IBM blockchain history for an individual item, making every single step from cradle to gate traceable, and calculate the carbon footprint associated with the overall production process [38].

### 2.7. COFCO Cooperation Ltd.

The company China Oil & Foodstuffs Corporation Cooperation Ltd. (COFCO), located in Beijing, PR China, is reported as the largest food and beverage producer in the People’s Republic of China. In addition, the company is a significant producer of biobased PLA polymers produced by their PLAneo^TM^ technology. The plant was engineered and constructed by ThyssenKrupp Industrial Solutions and started operating in 2018 [39]. Since then, COFCO has also commercialized P(3HB) homopolyester using extremophilic production strains, many of which are genetically modified. This production concept, named the Next Generation Biotechnology (NGIB), uses robust, extremophilic production strains such as recombinant *Halomonas bluephagenesis* (originally termed *Halomonas* sp. TD01), which allows production under nonsterile conditions with continuous cultivation conditions from inexpensive feedstocks, thus saving fresh water and energy [40,41]. The current production capacity is estimated at 1000 t *per annum*. COFCO’s downstream processing for PHA recovery is further simplified since *Halomonas* spp. cells are easily disrupted by sodium dodecyl sulfate (SDS) washing and release PHA granules into the aqueous phase [42]. The technology used by COFCO was initially used by two other Chinese start-ups: PhaBuilder and Medpha. The P(3HB) produced is being used for the production of textile fibers (personal communication by George Chen) [43]. The company has also filed a patent application in cooperation with Tsinghua University for reusing wastewater from the PHA production process using *Halomonas* sp. [44] (Chinese patent 202010358327.1, publication date 30 June 2020).

### 2.8. Mango Materials

Mango Materials, a start-up led by three female entrepreneurs and located in California, produces P(3HB) under the trade name YOOP from crude biogas, a mixture of CH_4_, CO_2_, and H_2_S. The company is co-located at a biogas-producing wastewater treatment plant in the San Francisco Bay Area. Current weekly quantities of about 100 kg P(3HB) are being produced as disclosed by the company. The products are recommended for use in injection molding, fiber extrusion, and additive manufacturing (3D printing) [45], the last being one of the currently most emerging applications for PHA [46]. The P(3HB) production process constitutes a closed-loop system where spent PHA materials made of YOOP^TM^ PHA can undergo conversion to biogas, generating the raw material for the next PHA-production cycle. At the moment, the company is looking for launching partnerships with various biogas producers such as landfills, wastewater treatment plants, and agricultural waste composting facilities. Production strains appear to be randomly cultured wild-type methanotrophs, although this is not revealed by the company. According to Mango Materials, no sterilization is required due to the robust nature of the strain and the process [45]. P(3HB) homopolyester is produced as the sole product, which the company claims to biodegrade in about 6 weeks in a marine environment [45].

### 2.9. Bio-On

Bio-On, a company located near Bologna, Italy, produced P(3HB) and P(3HB-*co*-3HV) for different applications under the brand name Minerv^TM^. They used beet sucrose, by-products of beet sugar production (molasses), and other agro-industrial surplus materials and claimed an annual production of approximately 2000 t of PHA starting in 2018. Bio-On mentions using *C. necator* as their production strain on their homepage. They claim to use a downstream process for PHA recovery from the non-PHA cell mass without using organic solvents for PHA extraction. On its internet site, the company claims to possess “the world´s largest PHA production plant” [47]. However, Bio-On came under severe financial trouble in 2019, declared bankruptcy in order to avoid liquidation, and continued operations under a temporary court order [48]. Their current status and future remain unclear.

Bio-On claims to have developed and filed a patent application for its Minerv products in PHA nanoparticles to diagnose and treat cancer (Minerv BIOMEDS^TM^). These nanoparticles simultaneously contained two different contrast media, namely magnetic nanoparticles and gold nanocylinders, which can rapidly mark tumor mass by NMR. Moreover, such nanoparticles could additionally contain chemotherapeutics to combat tumors [49]. In the bioremediation field, Bio-On announced in 2017 that tests were successfully carried out for biological degradation of oil slicks on sea surface; here, PHA powder was put on the oil film and was used as feedstocks for different microorganisms which are capable of hydrocarbon degradation [50]. In cosmetics and personal care, Bio-On Minerv^TM^ PHA was used as microbeads in emulsions for body and face peelings, cleaning agents, and toothpaste. The company claims that these products entered the market as “Minerv Bio-Cosmetics^TM^” replacing primary microplastic particles made of PE. The lipophilic and hydrophobic nature of P(3HB) gives cosmetic products a creamy texture at 5 to 20 µm particle sizes, which makes such PHA microparticles particularly interesting for skin care applications, where they do not remove water from the skin, but give oily skin a natural matt appearance [51]. In addition, PHA microparticles scatter light and can also be used as sunscreens, which Bio-On claims to have marketed together with Unilever (brand name “MyKAI^TM^”) [52]. In 2018, the company started marketing their so-called “Minerv Supertoys^TM^”, a form of biodegradable LEGO^TM^-like toy bricks made of colored Minerv PHA [53]. In 2019, Bio-On filed for a patent application for a cigarette filter containing a liquid-like P(3HB) product of PHA to replace triacetin. The patent application claims that this new filter system can retain up to 60% of dangerous reactive oxidative species from tobacco smoke [54]. Moreover, Bio-On also claims to have produced prototypes of various furniture pieces consisting of Bio-On PHA for the designer Kartell [55].

## 3. Industrializing P(3HB-*co*-3HV) Copolyesters

### 3.1. PHA Heteropolyesters and Their Advantages in Processing and Commercialization

P(3HB) homopolyester was considered to be the only PHA produced in nature since its discovery in the 1920s by Maurice Lemoigne [56] until Wallen and Davis isolated P(3HB-*co*-3HV) from dried activated sludge in the 1970s. They used chloroform to dissolve the PHA and precipitated it using ether and cooling the mixture. They then treated the resulting PHA samples with hot ethanol. The ethanol-insoluble fraction had a Fourier transform infrared spectroscopy (FTIR) spectrum and melting temperature (170 °C) of P(3HB) homopolyester, while the ethanol-soluble fraction precipitated when cooling down and had a melting point of 100–105 °C and a structure different from P(3HB) homopolyester [57]. Further research followed, and in 1984 Wallen and Rohwedder reported on the isolation of new microbial strains accumulating PHA from effluent water. Again, the properties of the PHA produced by these microbes were considerably different from the properties known from P(3HB) homopolyester; a *T_m_* below 100 °C or solubility even in cold ethanol were findings surprising at that time. Based on GC-MS analyses, it was confirmed that the polymer contained, besides 3-hydroxybutyrate (3HB), also 3-hydroxyvalerate (3HV) units and, to a minor extent, 3-hydroxyhexanoate (3HHx). This was the first unambiguous identification of PHA building blocks different from 3HB [58]. Using techniques such as gas chromatography–mass spectroscopy (GC-MS), Findlay and White discovered 11 additional and different 3-hydroxyalkanoates (3HAs) from marine sediments and 6 more in *Bacillus megaterium* PHA in 1983 [59]. In 1981, Morikawa and Marchessault discovered that pyrolysis of 3HB- and 3HV-containing microbial PHA generates unsaturated compounds (crotonic acid and pentenoic acid, respectively), which were recognized as valuable chemical synthons [60].

The fact that P(3HB-*co*-3HV) copolyesters can be produced by feeding appropriate 3HV precursors was subsequently discovered and patented by Holmes, Wright, and Collins for Imperial Chemical Industry Biological (ICI) in the early 1980s (EP0052459A1), where they claimed that “*The copolymers are made microbiologically: for part of the cultivation the micro-organism is under conditions of limitation of a nutrient, e.g., nitrogen source, required for growth but not polyester accumulation. For at least part of this period of growth limitation the substrate is an acid or a salt thereof that gives the comonomer units. Propionic acid, which gives polymers where n = 1, R<2> = R<3> = R<4> = H and R<1> = C_2_H_5_, is the preferred acid.*” [61].

In 1992, a seminal paper by Luzier summarized for the first time the advantages of P(3HB-*co*-3HV) copolyesters in comparison to P(3HB) homopolyester by comparing thermal and mechanical properties for different grades of PHA commercially produced that time by ICI (see next section); P(3HB) homopolyester and P(3HB-*co*-3HV) with 10 or 20 mol-% 3HV were studied. With increasing 3HV content, melting point *T_m_* decreased (170 °C for P(3HB) and 140 and 130 °C for the two P(3HB-*co*-3HV) grades, degree of crystallinity (80, 60, and 35%), tensile strength (40, 25, and 20 MPa), flexural modulus (3.5, 1.2, and 0.8 GPa), elongation at break (8, 20, and 50%), and impact strength (60, 110, and 350 J/m)). The lower *T_m_* of the P(3HB-*co*-3HV) copolyesters broadened their processing windows, making them suitable especially for extrusion, injection, and blow molding, which enabled the application of the materials for injection-molded parts, bottles, extruded sheets, films, fibers, and P(3HB-*co*-3HV)-coated paper [62].

### 3.2. ICI–Zeneca–Metabolix–Telles

Imperial Chemical Industry Biological (ICI), London, UK, started P(3HB-*co*-3HV) production as a reaction to the sudden oil shock of the 1970s. In this process, *C. necator* (in the original patent: *Alcaligenes eutrophus* NC 1B) was used as production strain. Cultivations were performed on the main substrate glucose (formation of biomass, 3HB, and energy) and propionic acid (precursor for 3HV) up to cell concentrations exceeding 100 g/L, with P(3HB-*co*-3HV) fractions in cell dry mass reaching up to 80 wt.-%. Phosphate-limitation acted as the factor to provoke the switch from the phase of balanced biomass growth to the phase of PHA biosynthesis in the ICI process. Importantly, for economic and environmental reasons, the company soon switched from solvent extraction for P(3HB-*co*-3HV) recovery to the use of surfactants and enzyme cocktails to disintegrate non-PHA biomass for release of the intracellular product. A limited number of copolymers having a different percentage of 3HV fraction were synthesized and obtained as a white powder. Based on customer needs, ICI then blended the various copolymer grades along with other additives to obtain the appropriate PHA compound granulates for sale to their customers. ICI started its commercial production plant that had the capacity to produce 5000 t annually and sold the products under the trade name BIOPOL^TM^ (in the USA under the trade name PHBV^TM^) at USD 7–8 per kilogram. Shampoo bottles (by the company Wella) and disposable razors were some of the products made with these P(3HB-*co*-3HV) compounds and marketed. Da Cruz Pradella estimated that in 1990 the demand for these compounds was between 5000 and 10,000 t per year [20,63].

In 1993, ICI spun off its agricultural and pharma division, including BIOPOL^TM^, as Zeneca Ltd. Zeneca, in turn, sold all its PHA-related patents and technology to Monsanto in 1996; it was estimated that, at that time, Zeneca had a production capacity of 600–800 t per year [5,20,63]. Monsanto, however, was more interested in producing PHA in transgenic plants, a process that has not been realized on a commercial scale as of this writing. Monsanto eventually ceased BIOPOL^TM^ production in 1999 and subsequently sold the entire ICI/Zeneca BIOPOL technology to Metabolix (based in Cambridge, Massachusetts) in 2001. Metabolix finally entered into the joint venture called Telles with Archer Daniels Midland (ADM) in 2006; Telles pursued the ambitious goal of commercializing P(3HB-*co*-3HV) under the trade name Mirel^TM^. Telles launched a production plant with an annual capacity of 50,000 t in 2009. Two types of P(3HB-*co*-3HV) were produced: a grade suitable for thermoforming and injection molding (Mirel^TM^ 3000 series) and a paper coating grade (Mirel^TM^ 4000 series) [64]. Compression molding grade Mirel^TM^ P1003 was produced through “*blends of poly(R-3-hydroxybutyric acid), poly(3-hydroxybutyrate-co-4-hydroxybutyrate), proprietary mineral fillers, and proprietary biodegradable additives*” [65]. Envisaged applications included shopping bags, compost bags, packaging, agriculture/horticulture films, aquatic applications, and production of various durable consumer goods. The process was based on sugars such as dextrose from hydrolyzed corn growing adjacent to the Telles fermentation plant in Clinton, Iowa [64]. ADM finally abandoned the joint venture in January 2012, which at that time was a real setback for the budding PHA industry and the numerous ambitious PHA research groups [66]. However, Metabolix carried on its activities in the biosynthesis of P(3HB) in recombinant oil plants and switchgrass, eventually developing PHA copolymers of P(3HB) and P(4HB), or P(3HB-*co*-4HB), including those that were elastomeric and had a high 4-hydroxybutyrate (4HB) co-monomer fraction (~ 50%) as reported later [42,67].

### 3.3. PHB Industrial S.A., Brazil

PHB Industrial S.A. (PHB/ISA), in Serrana, Brazil, also produces P(3HB-*co*-3HV) copolyesters from hydrolyzed sucrose from sugarcane, in addition to P(3HB) production already described above. Different 3HV-related precursors (propionic acid, valeric acid) were used to produce the copolymers [68]. PHB/ISA’s P(3HB-*co*-3HV), sold under the labels BIOCYCLE PHBV7 and BIOCYCLE PHBV19 contains 7 and 19 mol-% 3HV, respectively [31]. The BIOCYCLE^TM^ materials are certified as compostable according to DIN CERTO and Vinçotte [38]. Efficient copolyester production using this process may require a switch to alternative production strains different from *C. necator*, such as *Burkholderia sacchari* (wild-type isolate: IPT 101) and its mutants (strain IPT 189), allowing beneficial features such as direct utilization of sucrose, high specific growth rates above 0.4 h^−1^, and high volumetric productivity [69]. *B. sacchari* can also be used to produce block P(3HB-*co*-3HV) copolyesters using xylose and levulinic acid, both inexpensive starting materials originating from bagasse, a by-product of cane sugar production [70]. As shown in 2015 by Kovalcik et al., thermoforming of BIOCYCLE P(3HB-*co*-3HV) produced by the company PHB/ISA with Kraft lignin resulted in highly compatible composites. In this study, lignin exerted a reinforcing effect on the copolyester and improved its rather modest thermo-oxidative stability during melt processing, a typical problem when processing P(3HB) homopolyester and P(3HB-*co*-3HV) copolyesters with 3HV contents up to 15 mol-%. The new composite materials were shown to drastically increase the biopolyester´s barrier performance against O_2_ and CO_2_, which makes such biocomposites intriguing materials for packaging films for perishable food [71].

### 3.4. Tianan Biologic Materials Co.

Currently, Tianan Biologic Materials Co. in Ningbo, PR China, is known as “the world´s largest producer” of P(3HB-*co*-3HV) copolyesters with a reported production capacity of 2000 annual t [33]. These copolyesters with typically rather low fractions of 3HV (1–3 mol-%) are sold under the trade names ENMAT Y1000^TM^ (powder form), ENMAT Y1000P^TM^ (pellet form), and ENMAT Y1010^TM^ (processed with “nucleating and stabilizing agent”). Some researchers have also reported higher 3HV content of 5 mol-% [72,73] and 8 mol-% [74] in ENMAT^TM^ P(3HB-*co*-3HV), although the authors of the present review have no evidence of such grades being sold at present. These Tianan materials are used for producing a broad range of different marketable items via injection molding, thermoforming, and melt extrusion, with the melting temperature being slightly lower than that of the P(3HB) homopolyester ENMAT Y3000P^TM^. The company recommends detailed conditions for processing their materials on established fossil plastic processing machines. ENMAT^TM^ P(3HB-*co*-3HV) copolyesters have compostability certification (US Biodegradable Products Institute) and are listed as FCM substance No. 744 in Table 1 of Annex I of the Plastics Regulation of the European Union. They have also been EU REACH compliant since 2008 [32]. ENMAT^TM^ P(3HB-*co*-3HV) copolyesters can be used for film blowing due to their higher flexibility [75]. Overall improvement in processability and improvements in flexural strength, elastic modulus, and thermal stability of ENMAT^TM^ P(3HB-*co*-3HV) can be achieved by compounding them with various fractions of PLA [72] or with modified lignin [74]. Tianan also promotes PHA grades for denitrification of soil and in wastewater treatment plants and for spinning fiber and producing nonwovens [23,76]. Recently, biocomposites of ENMAT Y1000P^TM^ and Miscanthus fibers and distillers’ dried grains with solubles (DDGS) were prepared by Meereboer et al. [77] to further improve the marine degradability of the biopolyester. P(3HB-*co*-3HV) processed with Miscanthus showed 15 and 25% better biodegradability, respectively, compared to pure P(3HB-*co*-3HV). Compared to neat P(3HB-*co*-3HV), 85/15 and 75/25 composites of P(3HB-*co*-3HV) and DDGS showed 17 and 40% better biodegradability, respectively. The 75/25 P(3HB-*co*-3HV)/Miscanthus and 75/25 P(3HB-*co*-3HV)/DDGS biocomposites completely biodegraded in marine environments within 412 and 295 days, respectively [77].

### 3.5. Genecis Bioindustries Inc.

Genecis is one of the newest start-ups in PHA, producing their P(3HB-*co*-3HV) in Ontario, Canada. Genecis uses “discarded organic food waste” as raw material for its acidogenic bacterial colony to produce fatty acids. These fatty acids, in turn, are converted by PHA-producing microbes to P(3HB-*co*-3HV). All microbes used are reported as isolates from Canadian environmental samples. The process currently is carried out in a “large pilot bioreactor” at the University of Toronto, at the end of which they use “a chemical process to open the bacterial cells and extract the plastic particles”; the production strain(s) and capacity remain largely unrevealed. According to the company´s information, it is expected that 3 t of organic waste will be converted into P(3HB-*co*-3HV) per week at a new demonstration plant starting in 2021. Food packaging items such as cutlery, coffee pods, and 3D printing filaments are listed as possible fields of application for their PHA materials [78].

### 3.6. Bioextrax

In Sweden, Bioextrax AB, founded in 2014 as a spin-off from the biotechnology department of Lund University, produces PHA as granules at “a per kg price which is approximately 50% of other methods”. According to the company, PHA produced by the Bioextrax process is recovered via “*a universal, environmentally friendly and very cost-efficient recovery of PHAs from PHA producing bacteria*”; “*Bioextrax’s patented technology produces native-shaped bioplastics granules with intact molecular weight*” and can be applied to pure and mixed microbial cultures. This natural extraction process does without chemicals and solvents and leaves the nutrient-rich solubilized non-PHA biomass as a coproduct [79]. According to the underlying patent (WO2016085396A1), non-PHA biomass of the PHA producer is hydrolyzed by a *Bacillus pumilus* culture. At the moment, the company holds some patent-pending processes for the production of P(3HB-*co*-3HV) copolyesters and poly(3-hydroxyoctanoate) (P(3HO)) homopolyester, with production strains being kept confidential by the company. The company´s business model is to license the entire technology (PHA production and downstream processing) to big industrial PHA producers (personal communication by Edvard Hall, CEO Bioextrax [80]).

## 4. Industrializing P(3HB-*co*-4HB) Copolyesters

### 4.1. 4-Hydroxybutyrate: An Achiral Building Block as Game Changer for PHA Properties

4-Hydroxybutyrate (4HB) is the only well-studied achiral monomer found in natural PHA. 4HB was first described as a PHA monomer by the team of Yoshiharu Doi in Japan, who discovered this novel monomer in PHA samples produced by two strains of *C. necator* when supplied with butyric acid along with the 4HB precursors 4-hydroxybutyric acid or 4-chlorobutyric acid, while feeding butyric acid alone resulted in accumulation of P(3HB) homopolyester. Depending on the feed composition and the strain selected, up to 49 mol-% 4HB was incorporated into the P(3HB-*co*-4HB) copolyesters. The presence of 4HB in PHA was confirmed by nuclear magnetic resonance (NMR) (both solid-state ^13^C NMR and solution NMR were applied), and a significant decrease in crystallinity at increasing 4HB fraction in P(3HB-*co*-4HB) was reported, with the 49 mol-% 4HB copolyester being almost completely amorphous without any detectable crystalline regions [81]. As has been comprehensively reviewed by Utsunomia et al., these copolyesters can range from being thermoplastic to completely elastomeric depending on the 4HB fraction [82].

Doi´s group carried out an in-depth investigation on the properties of this novel class of PHA using X-ray diffraction and differential scanning calorimetry (DSC), revealing that the integration of 4HB building blocks into the highly crystalline P(3HB) matrix causes a considerably stronger drop in lattice crystallinity than exhibited by 3HV fractions in P(3HB-*co*-3HV) copolyesters [83]. Later, the impact of different 4HB fractions in P(3HB-*co*-4HB) copolyesters on *T_m_*, *T_g_*, and storage modulus (E′) was studied on compression-molded sheets, showing that all of these parameters decreased with increasing 4HB fraction. Yield stress and stress at break decreased only slightly with increasing 4HB content, while the elongation at break increased substantially, making such copolyesters highly flexible materials. Moreover, improved thermal stability was observed for melt-processed P(3HB-*co*-4HB) copolyester sheets with increased 4HB fractions [84].

### 4.2. Tianjin GreenBio Materials Co. Ltd.

Tianjin GreenBio Materials Co. Ltd. (GreenBio) in PR China was one of two companies that produced P(3HB-*co*-4HB). In 2009, GreenBio received a $20 million investment from the strain collection *Deutsche Stammsammlung für Mikroorganismen und Zellkulturen* (DSM) to scale their PHA production from pilot- to industrial-scale bioreactors of 150 m^3^ volume [85]. Recombinant *Escherichia coli* was used as the production strain, and glucose was used as the primary carbon source along with 1,4-butanediol to produce the copolyesters. GreenBio used an organic solvent-based downstream process to recover the PHA copolymers followed by washing with water to remove the organic solvents. This complex downstream process was one of the primary causes for the high price of the PHA, sold under the trade name SoGreen^TM^. Different grades of SoGreen^TM^ were labeled as SoGreen-00X^TM^, with “X” ranging from A to D (depending on 4HB fraction), all of them having a similar thermal decomposition temperature of about 286 to 290 °C. According to the company´s information disclosed online and in literature reports, the following SoGreen^TM^ grades were commercially available: SoGreen-00A-1^TM^ (3% 4HB), SoGreen-00B (12% 4HB), SoGreen-00C^TM^ (16% 4HB), and SoGreen-00D^TM^ (64% 4HB). SoGreen-00A-1^TM^ had a *T_m_* of 166 °C (manufacturer information [86]), and 150 °C according to reference [86]. SoGreen-00D^TM^ had an outstandingly low *T_m_* of 50 °C (manufacturer information) [85]. The standard material SoGreen-00B^TM^ (12% 4HB) had a *T_m_* of 125 °C (manufacturer information) [85], or 126 °C [87], while, surprisingly, SoGreen-00D^TM^ was reported to have a comparatively high *T_m_* of 152 °C [85]. Glass transition *T_g_* decreased with increasing 4HB fraction (SoGreen-00A-1^TM^: +4 °C [86], SoGreen-00C^TM^: −8 °C [85]). Predictably, elongation at break drastically increased from 10% (SoGreen-00A^TM^) to 775% (SoGreen-00D^TM^) with increasing 4HB content (manufacturer information) [85]. GreenBio reportedly had a PHA production capacity of 10,000 t per year, and the company claimed it as the world´s largest PHA production facility in 2021 [85]. Besides selling the pure P(3HB-*co*-4HB) resins, Tianjin Green Bioscience also modified their SoGreen^TM^ products with undisclosed additives for several different applications; pellets for film blowing were sold as SoGreen 2013^TM^ with an elongation at break of 174–180% and a tensile strength of 41–45 MPa for applications such as “fresh film, mulching film, laminating film, wrapping film, heat-shrinkable film, food packaging, shopping bags, garbage bags, gift bags, produce bags” (manufacturer information). Pellets for foam applications were sold as SoGreen 1023^TM^ for expandable food-service ware, producing mesh foam bags for fruits, cushion pads, cushion fillers, etc. They had a tensile strength of 35 MPa, elongation at break of 300%, a flexural modulus of 1200 MPa, and a flexural strength of 48 MPa. Pellets for production of sheets, boards, and injection-molding products were commercialized as SoGreen 3001^TM^. Mechanical properties of these products significantly differ from the more flexible products SoGreen 2013^TM^ and 1023^TM^: SoGreen 3001^TM^ had a low tensile strength of 21 MPa and an elongation at break of only 42%, and it had lower flexural modulus and flexural strength of 25 MPa and 960 MPa, respectively [85].

However, GreenBio came under severe economic pressure [42]. GreenBio products did not perform as expected due to the insufficient market demand due to high product prices, which in turn was the result of high production costs, and it stopped its production about two years ago (personal communication by George Chen [43]).

### 4.3. Shenzhen Ecomann Biotechnology Co. Ltd.

Another Chinese P(3HB-*co*-4HB) manufacturer was Shenzhen Ecomann Biotechnology Co. Ltd., established in 2008 and located in Shenzhen, Guangdong. They processed their AmBio^TM^ P(3HB-*co*-4HB) into customer-ready products like “aquarium biopellet filter media” (about 5000 t annual capacity, selling price ca. USD 14) and resins for film blowing (annual capacity of 5000 T; selling price 3.8–4.0 USD/kg). They also commercialized 500 t of PHA per year for biodegradable dog bags at a price below USD 0.01 per piece, and 500 t per year of PHA for the “Compostable and Biodegradable Green Bag” for shopping (USD 0.03 per piece), biodegradable bags for household and kitchen waste (“bin liners”; USD 0.02 per piece), mulch films made of PHA, biodegradable iPhone cases, and PHA 3D printing inks and filaments. All these bags were labeled “OK compost” (TÜV, Austria), indicating their home compostability [88]. The Ecomann grades of P(3HB-*co*-4HB) were labeled based on 4HB fraction in them such as EM 40000^TM^ (3HB:4HB = 13:1), EM 20010^TM^ (3HB:4HB = 18:1), and EM 10070^TM^ (3HB:4HB = 10:1). Some blends were also sold containing PLA and fillers. Products such as EM 30000^TM^ and EM 50000^TM^ were also marketed, but their 4HB content was not reported. *T_m_* values for these materials were between 140 and 160 °C (110–130 °C for EM 50000^TM^). Tensile strength was reported to be 41–49 MPa (EM 40000^TM^) and 38–40 MPa (E 20010^TM^), and degrees of crystallinity were around 15–16%. All materials were “OK compost HOME” certified and FDA approved. Raw Ecomann PHA was also sold as raw material to buyers such as BASF in China [88].

In a study by Coltelli et al. [89], Ecomann EM 5400 F^TM^ was used to prepare skin-compatible beauty masks by blending with starch and embedding bioactive compounds to be released to the skin under wet conditions. Calcium carbonate microparticles were added to control stickiness during molding, and their films were manufactured via compression molding. The prepared films were tested and turned out to be highly biocompatible in in vitro experiments. The authors blended Ecomann PHA with other biopolyesters such as PBAT, poly(butylene succinate-*co*-butylene adipate) (PBSA), and starch and prepared novel beauty masks. The ternary blends showed better processability during melt extrusion at 140 °C [89]. Their primary goal in going after their applications was to target the beauty care market, which they determined to be more than USD 5 × 10^11^ in 2017, with estimates of up to USD 8 × 10^11^ in 2025, a potentially large and important market opportunity for biocompatible and biodegradable PHA. The Ecomann P(3HB-*co*-4HB) grades were also tested in the biomedical field by Wu et al. [90], who prepared compression-molded membranes of this material after mixing it with chitosan in a Brabender. These materials showed high biocompatibility in a cytocompatibility test, in addition to antimicrobial activity which varied with the chitosan content, making them viable for wound dressing applications [90].

### 4.4. Metabolix and Cheiljedang Corporation

After the dissolution of the joint venture Telles, set up between ADM and Metabolix, Metabolix focused its attention on producing P(3HB-*co*-4HB) copolymers using recombinant *E. coli*. They already had experience in producing P(4HB) homopolymers which Tepha, a spin-out from Metabolix, uses in its products. Metabolix developed and produced a wide variety of P(3HB-*co*-4HB) copolymers starting from semicrystalline P(3HB-*co*-4HB) (5–15% 4HB; *T_g_* −10 °C, *T_m_* 119 °C, tensile strength 36 MPa) to amorphous P(3HB-*co*-4HB) (up to 50% 4HB; *T_g_* −30 °C, tensile strength 1.5 MPa). The company claimed biodegradation of their products by 90% in marine environments after 120 days, depending on the 4HB fraction in the copolyesters and the thickness of the parts. Applications ranged from compostable shopping bags, rigid food containers, drinking straws, coffee capsules, mulching films, and fishing nets to formulations for controlled release of pesticides, for paper coating, and as 3D printing inks [91]. Most of the polymers were produced via contract manufacturing and no volume figures were released.

In 2016, Metabolix reorganized itself as Yield10 Biosciences, an agricultural bioscience company, led by Oliver Peoples, CEO, and Kristi Snell, CSO, both pioneers in PHA. Yield10 continues to develop PHA in camelina plants which they believe would allow them to reach price points similar to commodity plastics. In January 2021, Yield10 disclosed their successful field tests of prototype lines of the recombinant oil plant *Camelina sativa* engineered to accumulate PHA biopolymers (presumably P(3HB) homopolyester) directly in seeds, with PHA fractions in seeds reaching about 6%. For 2021, large field tests were envisaged, provided that regulatory approval by the authorities will be given [92,93].

The assets of Metabolix’s microbial production of PHA along with the intellectual property were sold to CJ Cheiljedang, and CJ renamed the commercial entity CJ White Bio. CJ White Bio has recently reaffirmed their commitment to PHA and to the grades that Metabolix had come up with in many of the same markets. They have announced the establishment of a 5000 ton per year plant in Indonesia to produce P(3HB-*co*-4HB) [42,92].

### 4.5. PhaBuilder and Medpha

Several start-up companies were founded during the last several years in PR China to manufacture P(3HB-*co*-4HB) using the NGIB concept, similar to the COFCO process for P(3HB) homopolyester production, which has been mentioned above [39]. Among these companies, PhaBuilder produces P(3HB-*co*-4HB) by recombinant *Halomonas* ssp., a recombinant of the halophilic Chinese salt lake isolate *Halomonas* sp. TD01 (later: *Halomonas bluephagenesis* [94]), on an annual production scale of several tons [42]. They used a recombinant version of the strain *H. bluephagenesis* TD40, which was cultivated on a 5 m^3^ scale on glucose, corn steep liquor, and γ-butyrolactone (GBL) already in 2018. They reported to have obtained 100 g/L dry biomass containing 60.4% P(3HB-*co*-13.5 mol-%-4HB) at a productivity of about 1.7 g/(L·h) [95]; the industrial process still uses the same feedstocks as described in this article (personal communication with George Chen) [43]. PhaBuilder´s standard P(3HB-*co*-4HB), labeled “mP34HB 10”, is a fully biodegradable and semicrystalline material. After granulation (prepared by blending with plasticizers, heat stabilizers, and processing additives), it can be used for film blowing, extrusion, injection molding, and 3D printing. mP34HB 10 is reported to biodegrade in different natural environments tested, such as seawater, freshwater, sewage, sludge, and soil, and undergoes composting. mP34HB 10 meets the requirements of biodegradation standards ASTM 6400 and EN13432. The material is also widely used in polymer modification with other compatible biobased materials such as PLA, other aliphatic polyesters (such as poly(butylene succinate) (PBS), PBSA, or PBAT), and thermoplastic starch (TPS), in addition to some non-biodegradable materials (such as TPU, PVAc, POM, PMMA, and ABS) [96].

PhaBuilder also plans to produce other types of PHA such as P(3HB), P(3HB-*co*-3HHx), and P(3HB-*co*-3HV). In addition, the company carries out extensive research for new applications of the 3HB monomer [42].

Medpha also produces P(3HB-*co*-4HB) biopolyesters on an industrial scale using the recombinant *Halomonas* ssp. originally derived from *Halomonas* TD01; as the name indicates, this company concentrates on producing PHA explicitly for medical applications (personal communication with George Chen) [43].

### 4.6. Tepha Medical Devices Inc.

The linear, pliable homopolyester P(4HB) has remarkable material characteristics, which are completely different from those observed for the above-described short-chain-length (*scl*)-PHAs, such as P(3HB) and P(3HB-*co*-3HV). Most of all, P(4HB) has an exceptionally high elongation at break of up to 1000%, which makes it enormously stretchable and flexible. This compares favorably with other polymers such as PLA, poly(glycolic acid) (PGA), or P(3HB), which has an elongation at break of only about 3–7% [97,98], and poly(ε-caprolactone) (PCL), which has an elongation at break of about 60% [99]. Oriented P(4HB) fibers have tensile strength values of about 545 MPa, which is higher than for poly(propylene) (PP) sutures (410–460 MPa), making them very interesting and viable as biological fiber applications such as sutures, although P(4HB) sutures have a significantly lower Young’s modulus than other marketed monofilament sutures [100].

In 2001, Metabolix Inc., Cambridge, USA, filed a patent for the production of P(4HB) and 4HB-rich copolyesters by using stable recombinant strains such as *E. coli* from inexpensive feedstocks. In 2007, the company Tepha Inc., USA, started, based on this patent, producing a range of PHA-based biomedical products in Lexington, MA, USA, e.g., P(4HB)-made TephaFLEX^TM^ sutures, which are US Food and Drug Administration (FDA) approved in 2007 (notably, it is still the only PHA approved for biomedical application to date!) [82]. In addition, the entire production process is ISO 13485 compliant, referring to the quality management for manufacturing of medical devices; this also includes a patented downstream processing for yielding an “extremely high purity material” by a solvent–antisolvent process (US9480780B2). Tepha holds a high number of patents on production, processing, and application of their material P(4HB) homopolyester, which is produced using an engineered and recombinant *E. coli* K12 production strain. Generally, multifilament fibers, multifilament meshes, polymer tubing, and thin films can be produced from TephaFLEX^TM^ products. A range of surgical materials based on P(4HB) or P(3HB-*co*-4HB) (trademark TephaELAST^TM^), e.g., absorbable meshes (for hernia repair, wound support, tissue reinforcement), threads, or films for a variety of surgical procedures, are produced by Tepha Inc., Lexington, MA, USA, [101], as are textile fibers for weaving, knitting, and braiding in cooperation with big textile manufacturers [102].

## 5. Industrializing P(3HB-*co*-3HHx) Copolyesters

### 5.1. Hybrid-Type PHA Copolyesters Consisting of Scl- and Mcl-PHA Building Blocks

Hybrid *scl-mcl*-PHA copolyesters consisting of 3HB and small amounts of medium-chain-length (*mcl*)-PHA building blocks (3HHx, 3-hydroxyoctanoate (3HO), 3-hydroxydecanoate (3HD)) were originally developed and patented by Isao Noda and coworkers. This Nodax^TM^ type of PHA helps to overcome problems associated with well-established P(3HB-*co*-3HV) copolyesters, namely the “isodimorphism”, where 3HV units can be easily incorporated into the crystal 3HB-lattice and *vice versa*, thus preventing disruption of the highly crystalline matrix. *Mcl*-building blocks, in contrast, disturb the 3HB matrix even more efficiently than the achiral building block 4HB does. In particular, P(3HB-*co*-3HHx) with a 3HHx content of 10–17 mol-% features excellent flexibility, demonstrated through exceptionally high elongation at break of up to 850%, which outperforms commercially available P(3HB-*co*-3HV) with 20 mol-% 3HV [103]. Indeed, this group of PHAs currently holds great promise for a permanent industrial-scale production by different companies.

The first report on the production of such hybrid-type P(3HB-*co*-3HHx) copolyesters was provided by Kobayashi et al. [104], who showed production of poly(3-hydroxybutyrate-*co*-3-hydroxyhexanoate) (P(3HB-*co*-3HHx)) by *Aeromonas* spp. on fats and oils. This was a real scientific surprise at the time; before that, it became widely accepted that microbes produce either *scl*-PHA or *mcl*-PHA, simply depending on the type of PHA synthase active in a given production strain. These novel findings resulted in the first patent on such type of PHA by Shiotani and Kobayashi for Kanegafuchi (Kaneka) Chemical Industry Co. Ltd. (U.S. Patent 5,292,860, 1994), which already claimed the use of *Aeromonas caviae* as production strain. Later, patents for production of this type of PHA were filed by Procter & Gamble for the inventions in this field by Isao Noda (U.S. Patent 5,498,692, 1996; U.S. Patent 5,990,271, 1999), in addition to a patent on halogen-free processes for recovery for such *scl-mcl*-PHA copolyesters (U.S. Patent 5,942,597).

On a larger scale, such materials were for the first time produced by wild-type strain *Aeromonas hydrophila* cultivated on glucose during the balanced growth phase, followed by feeding lauric acid under phosphate limitation during the PHA accumulation phase. This strain was isolated from raw sewage samples by Sang Yup Lee´s group and turned out to produce P(3HB-*co*-3HHx) from lauric acid and oleic acid [105]. The large-scale process was described by Chen et al., who achieved a volumetric productivity for P(3HB-*co*-3HHx) containing 11 mol-% 3HHx of 0.54 g/(L·h) in a two-stage batch process on a 20 m^3^ scale, corresponding to a production of 100 g/L biomass containing 50% P(3HB-*co*-3HHx) within 46 h of cultivation [103]. Importantly, the authors underlined in this study that a typically low-productive batch cultivation mode was applied due to complications observed when running this process with *A. hydrophila* 4AK4 in fed-batch mode.

Besides wild-type *A. caviae*, genetically modified *Ralstonia eutropha* (today: *C. necator*) expressing the *Pseudomonas fluorescens* GK-13 synthase gene is also reported to accumulate *scl-mcl*-PHA hybrid copolyesters; dependent on the substrate provided (salts of different fatty acids), the generated copolyesters contained, besides 3HB, building blocks such as 3HHx, 3HO, 3HD, or 3HDD [106].

### 5.2. Danimer Scientific

Danimer Scientific was founded in 2004 to manufacture biobased polymers and their compounds with, for example, PLA and PHA. In 2007, Danimer Scientific purchased the assets of the Nodax^TM^ PHA biopolymers from Proctor & Gamble (P&G), Cincinnati, OH, USA. P&G had by then developed and industrially produced P(3HB-*co*-3HHx) biopolymers under the Nodax^TM^ tradename based on their patents in the field. In 2013, Isao Noda joined DaniMer/Meredian Inc. after spending three decades with P&G; today, he is a member of the Board of Directors in addition to his many other responsibilities within Danimer Scientific. In 2014, Danimer and Meredian Inc. merged to form Meredian Holdings Group (MHG), which was then renamed to Danimer Scientific in 2016 and now produces Nodax^TM^ P(3HB-*co*-3HHx) commercially in Bainbridge, Georgia, and Winchester, Kentucky. The standard materials contain 5% 3HHx (*T_m_* about 150 °C); sheets with 7.1% 3HHx (*T_m_* 112–129 °C) and flakes/pellets with 6.5% 3HHx (*T_m_* 121–146 °C) are also manufactured. Production is performed based on proprietary processes; not all details, e.g., bioreactor types, production strains, and downstream processing, are disclosed. Their production capacity is estimated at 10,000 t per year [20,107].

Recently, Danimer Scientific announced breaking ground in both Georgia and Kentucky for the construction of additional capacity in PHA production. A few months back, Danimer acquired the technology and assets of Novomer Inc., a start-up out of upstate New York. Novomer is reputed to have invented a pathway to produce poly(3-hydroxypropionate) (P(3HP)) polymers using a chemocatalytic pathway. P(3HP) is described to have excellent barrier properties. The beverage company Bacardi^©^ announced that they have partnered with Danimer Scientific to convert their poly(ethylene terephthalate) (PET) bottles into PHA-based bottles, thus reducing about 3000 annual t (corresponding to 80 million bottles) of PET currently used to bottle their alcoholic drinks [107].

Production of P(3HB-*co*-3HHx), a hybrid *scl-mcl*-PHA, by Danimer Scientific starts from bioconversion of natural oils such as from palm, canola, or soy. There is some discrepancy about the production strain used for this process: while some sources report “presumably *A. caviae*” as production strain [20], others refer to “recombinant *R. eutropha*” [42]. According to Bacardi^©^, these “100% biodegradable spirit bottles” will biodegrade within 18 months in industrial composting facilities, soil, freshwater, and marine environments [108]. In addition, according to Danimer´s homepage, a “*private-label manufacturer will use Danimer Scientific’s Nodax^TM^ PHA for marine degradable straws with plans to expand into adjacent product categories*”. This company appears to be Wincup^©^, which has now publicly announced the production and marketing of its PHA-based straws. According to a press release from December 2020, Kemira, a pulp and paper chemicals company, is currently evaluating “Danimer Scientific’s Nodax™ PHA as commercial, fully biobased alternative for polyethylene coatings to manufacture recyclable paper and board products from renewable sources” [109].

### 5.3. Kanegafuchi Chemical Industry Co. Ltd. (Kaneka)

Kaneka, headquartered in Minato-ku, Tokyo, is an early developer and producer of PHA biopolymers. Their PHA biopolymer PHBH^TM^ is also P(3HB-*co*-3HHx), similar to Danimer Scientific, with differences in the 3HHx content in the final polymer. The above-mentioned patent (U.S. Patent 5,292,860, 1994) by Shiotani and Kobayashi for Kaneka, which claims the use of *Aeromonas* sp. for *scl*-*mcl*-PHA biosynthesis, forms the basis for Kaneka’s PHA biopolymer industrialization. It was later shared with Procter & Gamble, although the two companies P&G (and later Danimer Scientific) and Kaneka use different feedstocks, with Kaneka’s feedstock being palm oil and Danimer´s being Canola oil. Kaneka reportedly increased their production capacity to about 5000 t per year in 2020, an increase of about 5-fold from their previous capacity [110].

In Japan, approximately 10,000 Seven-Eleven Japan stores have started using PHBH^TM^ drinking straws for Seven Café^TM^ ice coffees since 2019 [111]. Moreover, in 2019, Kaneka launched the development of cosmetic containers with the company Shiseido Co. Ltd. (Tokyo, Japan); the product “AquaGel Lip Palette^TM^”, a biodegradable container for makeup, has been commercially available since the end of 2020 [112]. In addition, many global brand holders are studying a wide range of applications for PHBH^TM^ such as straws, plastic bags, cutlery, and food containers and packaging materials [110]. In addition, there is a project ongoing by Kaneka on teaching a polymer processing company in Kenya to produce PHBH^TM^ waste bin liners as a contribution to reducing the plastic waste problem in developing countries [113].

Currently, Kaneka is also commercializing other grades of PHBH^TM^ biopolymers such as rigid-grade X131A and X331N (6% 3HHx, 30% crystallinity) and semirigid grade X151A (11% 3HHx, about 5% crystallinity) [110]. Indeed, leading scientists from the PHA field such as Alexander Steinbüchel (University of Münster, Münster, Germany) currently consider the industrialization endeavors for PHA by Kaneka the probably most promising activities for a large-scale market entry of these products (“*I see only one technically reliable PHA product, the copolyester of 3HB and 3HHx produced by Kaneka in Japan, which is currently on the market.*”) [114].

### 5.4. Bluepha

**Figure 2:**
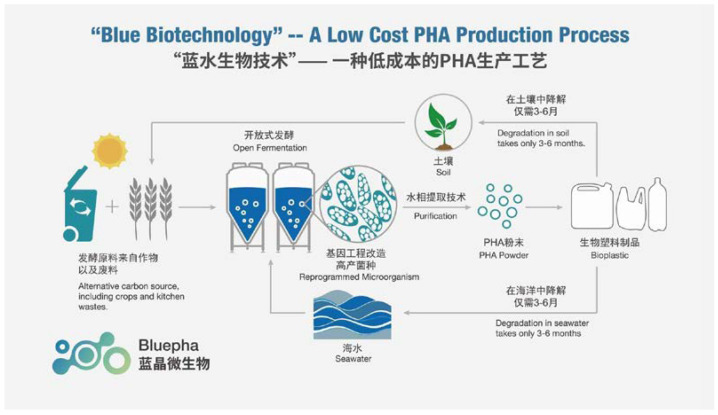
Illustration of the “Bluepha” process for industrial PHA production based on the principles of Next Generation Industrial Biotechnology (NGIB) [115]. The English and Chinese text are the same.

In the context of NGIB, the Chinese company Bluepha explicitly mentions on their internet site that “*plastics should be from nature and go back to nature*” [115]. This company uses a recombinant strain of the soil bacterium *C. necator* obtained by means of synthetic biology for production of P(3HB-*co*-3HHx) copolyesters (“*Bluepha is programmer of cells*”); a schematic of the process, presented by the company itself, is provided in Figure 2. The production scale of this plant is reported to be 1000 t annually [42]. According to the company, “alternative carbon sources” such as crops and kitchen waste are used as substrates for the cultivation, which is carried out in seawater. The Bluepha P(3HB-*co*-3HHx) is reported to be readily degraded both in seawater and soil within 3–6 months. The product is sold as pellets for injection molding, (aquarium) water restoration, textiles, and polymer films and as 3D-printing inks [115].

### 5.5. RWDC Industries Ltd.

The Singapore- and US-based company RWDC Industries Ltd. produces Solon^TM^ PHA by upcycling waste cooking oil [116]. As a primary market, the company plans to replace an impressive quantity of “1.4 trillion (*sic!*) drinking straws by 2025” (press release; quantity and the true need for so many straws must be questioned) [117]. Based on a previously established technology, P(3HB-*co*-3HHx) is produced by a recombinant *C. necator* strain; on their internet site, the company discloses a PHA fraction of in cell biomass of 80%. Their new 25–50 kt plant in Athens, Georgia, is announced to be fully operational by the second half of 2021. This US-based facility is planned to initially produce about 4000 t of Solon^TM^ per year, but the output is planned to increase to 350 kt by 2025, which would multiply the current global PHA productivity by more than 10 times. The company also plans to build more plants in Asia to meet the growing demand for biodegradable plastics in this region, which would consist of modular 25 kt production plants to be quickly installed at locations that are defined to be strategic to best serve customer demands. According to experts from the polymer industry, Solon^TM^ PHA needs to be sold at less than USD 4 per kg in order for RWDC to succeed in the market. Besides drinking straws, the company plans to enter the market for coffee cups and lids, cutlery, light-weight shopping bags, foods containers, and textile fibers. According to the company´s CCO, Blake Lindsey, “*The applications are endless and the impact is profound*.” [118].

## 6. Industrializing *Mcl*-PHA Copolyesters

Regarding the types of PHA biopolymers produced on an industrial scale, there is, to the best of the authors´ knowledge, only one company commercializing *mcl*-PHA, a group of PHA with building blocks having at least six carbon atoms, such as 3HHx and longer monomers, at any scale: PolyFerm Canada, located in Ontario, produces VersaMer^TM^ PHA as “irregular pieces, pellets, and latex”, encompassing, *inter alia*, P(3HO-*co*-3HHx), poly(3-hydroxyheptanoate-*co*-3-hydroxynonanoate) (P(3HHp-*co*-3HN)), and *mcl*-PHA containing also unsaturated building blocks. These materials have outstandingly low *T_g_* (−45 to −35 °C), low *T_m_* (45 to 65 °C), low molecular mass as is typical for *mcl*-PHA (100–150 kDa), and extraordinary high elongation at break of 1200–1400% [119]. In 2016, PolyFerm Canada licensed its technology to TerraVerdae Bioproducts, an industrial biotechnology company developing advanced bioplastics and biomaterials from C1-feedstocks. They are said to be scaling up *mcl*-PHA production based on personal communication with Bruce Ramsay, the cofounder of PolyFerm [120,121]. However, the majority of companies commercializing PHA are focusing on the “top-selling” *scl*-PHA products P(3HB), P(3HB-*co*-3HV), P(3HB-*co*-4HB), and P(3HB-*co*-3HHx), in addition to Tepha´s surgically important homopolyester P(4HB), as described in the previous sections.

## 7. Conclusions

As mentioned in the introduction, we are currently witnessing a significant wave of activities in PHA development and commercialization. While most of the commercial activities are focused on *scl*-PHA as bulk polymers such as P(3HB), P(3HB-*co*-3HV), P(3HB-*co*-4HB), and P(3HB-*co*-3HHx) and to a minor extent on P(4HB) and highly amorphous *mcl*-PHA copolyesters, most of these biopolymers are processed exclusively into single-use products. The next several years will draw a clearer picture about which concepts in the marketing of PHAs are indeed future-fit, be it in terms of market requirements, customer acceptance, sustainability, or economic feasibility. However, it is quite clear that the long-term success of commercial PHA needs to be grounded on some defined solid fundaments: robust and powerful microbial production strains, optimized and simple cultivation facilities, amply available renewable feedstocks, and sustainable and inexpensive downstream processing technologies, pointing to its ease of production and acceptable price points that would allow its proliferation to serve various markets

In any case, considering the currently already well-established status of biotechnological PHA manufacturing and the far-developed understanding of the metabolic background of PHA biosynthesis, we can expect that the current wave of commercialization of PHAs has indeed started. This is in contrast to the first commercialization efforts for PHAs made several decades ago: That time, we witnessed some short-term efforts and successes in biopolymer production during periods of crude oil shortage and exploding prices of fossil resources, but rapidly declining interest in renewable-sourced polymers, chemicals, and solvents as soon as the price for fossil resources dropped again. Now, the time is ripe for a continued market presence of these biopolymers. During the next few years, it will be of utmost interest to follow which types of PHA biopolymers, industrialization concepts, production strains, raw material sources, cultivation modes, and applications (single-use or durable) will prevail in the long term.

## Figures and Tables

**Table 1:** Most commercialized types of PHA (P(3HB), P(3HB-*co*-3HV), P(3HB-*co*-4HB), P(3HB-*co*-3HHx), P(4HB)), production strains, substrates, manufacturers, manufacturing scale/capacity, and certifications.

Type of PHA	Production Strains (Origin)	Substrates	Manufacturer	Logo	PHA Brand Name (Trade Mark)	Capacity (t/year)	Certifications/Approvals
**Poly(3-hydroxybutyrate)** 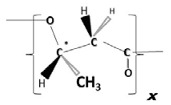	*Cupriavidus necator* (soil bacterium)	Glucose	ICI, London, UK (technology transferred to Zeneca, Monsanto, and finally Metabolix)	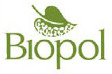	BIOPOL	Stopped (was about 800 in 1996)	-
		Hydrolyzed cane sugar	PHB Industrial S.A. (PHB/ISA), Serrana, Brazil	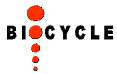	BIOCYCLE	~100 (entire PHA production capacity)	Compostable according to DIN CERTO and Vinçotte
		Beet sucrose and by-products of sugar beet industry (molasses) plus additional surplus products from agriculture	Bio-On, Bologna, Italy	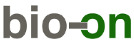	Minerv-PHA	2000 (current situation unclear!)	“Biodegradable”: according to USDA (“certified biobased product”) and TÜV Austria “OK biodegradable”; according to company: “MINERV-PHA™ dissolves in normal river or sea water leaving no residue in just a few days.”
	*Azohydromonas australica (Azohydromomas lata)* (soil bacterium)	Sucrose	Biomer, Schwalbach, Germany	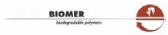	Biomer	900 (capacity)	“Fully biodegradable and compostable”
	*Paraburkholderia sacchari* (soil bacterium)	Sucrose	PHB Industrial S.A. (PHB/ISA), Serrana, Brazil	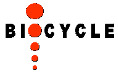	BIOCYCLE	~100 (entire PHA production capacity)	Compostable according to DIN CERTO and Vinçotte
	*Halomonas* sp. (*Halomonas bluephagenesis* ssp.) (salt lake isolate)	Presumably glucose	COFCO, Beijing, PR China	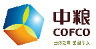	COFCO PHA	1000 (capacity)	n.r.
	Not disclosed Methanotroph (“robust strain”; origin n.r.)	Crude biogas (CH_4_, CO_2_, H_2_S)	Mango Materials, Redwood City, CA, USA	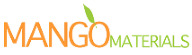	YOOP	0.25 (pilot scale; long-term goal: about 5 t per year)	“Fully biodegradable and compostable”
	“Newlight´s biocatalyst 9X” (marine isolate)	CH_4_ and CO_2_ from greenhouse gases	Newlight Technologies LLC, Huntington Beach, CA, USA	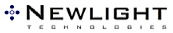	AirCarbon	n.r.	“Fully biodegradable”; “readily compostable”
	“Own microbiological collection”; wild-type organisms (origin not disclosed)	Waste cooking oil (Hydal technology)	Nafigate Corporation, Prague, Czech Republic	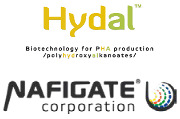	Hydal PHA	n.r.	FDA approved for food contact (FCN 1754), “carbon-negative” certified (ISO 14046-3 and specification for the assessment of the life cycle greenhouse gas emissions of goods and services (PAS 2050: 2008/2011)), “ocean degradable” (ASTM D6691 and D7081), “industrially compostable” (ASTM D6400)
**Poly(3-hydroxybutyrate-*co*-3-hydroxyvalerate)** 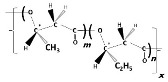	*Cupriavidus necator* (soil bacterium)	Glucose plus 3HV precursor	ICI, London, UK (technology transferred to Zeneca, Monsanto, and finally Metabolix)	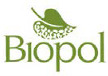	BIOPOL	Stopped (was about 600–800 in 1996)	-
		Glucose plus 3HV precursor	Telles (joint venture of Metabolix and ADM from 2009 to 2012)	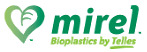	Mirel	50,000 (capacity in 2009; stopped in 2012)	n.r.
		Glucose plus 3HV precursor (glucose deriving from cassava starch)	Tianan Biologic Materials Co., Ningbo, PR China	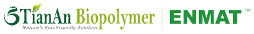	ENMAT	2000	“Compostable” according to US Biodegradable Products Institute (BPI) Food Contact Material (“FCM”) substance No. 744 in Table 1 of Annex I of the Plastics Regulation of the EU (REACH)
		Hydrolyzed cane sucrose plus propionate	PHB Industrial S.A. (PHB/ISA), Serrana, Brazil	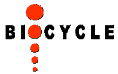	BIOCYCLE	~100 (entire PHA production capacity)	n.r.
		Beet sucrose and by-products of sugar beet industry (molasses) plus additional surplus products from agriculture plus 3HV precursors	Bio-On, Bologna, Italy	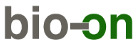	Minerv-PHA	2000 (current situation unclear!)	“Biodegradable”: according to USDA (“certified biobased product”) and TÜV Austria “OK biodegradable”; according to company: “MINERV-PHA™ dissolves in normal river or sea water leaving no residue in just a few days.”
	*Halomonas* sp. (*Halomonas bluephagenesis* ssp.) (salt lake isolate; genetically engineered)	Presumably glucose plus 3HV precursor	PhaBuilder, Beijing, PR China	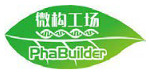	-	n.r.	n.r.
	*Haloferax mediterranei* (marine salt brine at Spanish coast)	Sugars, starch, glycerol (no 3HV-related precursors needed)	Not commercially produced yet, but high industrial potential supposed	-	-	-	-
**Poly(3-hydroxybutyrate-*co*-4-hydroxybutyrate)** 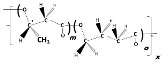	Rec. *Escherichia coli* (Enterobacterium)	Glucose plus 1,4-butanediole (4HB precursor)	Tianjin GreenBio Materials Co. Ltd., Tianjin, PR China	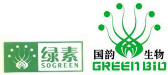	SoGreen	10,000	n.r.
			CJ, Seoul, Republic of Korea (technology from Metabolix)	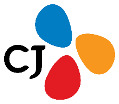	Yield10	n.r.	n.r.
		Not disclosed	Tepha Medical Devices Inc., Lexington, MA, USA	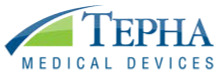	TephaELAST	n.r.	FDA approved for biomedical use as implant material; the entire production process is ISO 13485 compliant
	Not disclosed	Sugar plus 4HB-related precursor	Shenzhen Ecomann Biotechnology Co. Ltd., Guangdong, PR China	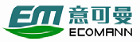	AmBio	10,000 (planned: 75,000 capacity)	“OK compost” “OK compost HOME” FDA approved
	*Halomonas* sp. TD40 (salt lake isolate)	Glucose, corn steep liquor, and GBL	PhaBuilder, Beijing, PR China	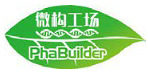	mP34HB 10	1000–10,000 (entire PHA production capacity)	Biodegradable according to ASTM 6400 and EN13432
	*Halomonas* sp. (*Halomonas bluephagenesis* ssp.; presumably strain TD40)	Glucose, corn steep liquor, and GBL	Medpha, Beijing, PR China	n.r.	Medpha PHA	100	n.r.
**Poly(3-hydroxybutyrate-*co*-3-hydroxyhexanoate)** 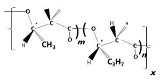	Presumably *Aeromonas caviae* or *Aeromonas hydrophila;* other sources (Tan et al., 2021) assume rec. *C. necator* (soil bacteria)	“Inexpensive oils derived from the seeds of plants such as canola and soy”	Danimer Scientific, Bainbridge, GA, USA (formerly Meredian Holdings Group Inc. and MHG; technology originally from Proctor & Gamble, Cincinatti, OH, USA)	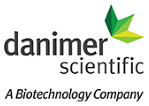	Nodax	10,000	Biobased (ASTM D6866; “OK biobased”); anaerobic and aerobic digestion in soil freshwater (“OK biodegradable SOIL”), freshwater (“OK biodegradable WATER”), marine environment (ASTM D6691), industrial and home composting (according to TÜV Austria and EN and ASTM norms). FDA approved for food contact
	Rec. *C. necator*	Vegetable oils	Kanegafuchi Chemical Industry Co. Ltd. (Kaneka), Tokyo, Japan	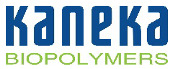			“OK compost INDUSTRIAL”, “OK compost HOME”, “OK biodegradable SOIL” (certification in progress), and “OK biobased” according to TÜV Austria; the “Biobased” certification for Japan; and the “Industrial Compostable” certification for Japan and USA
		Waste cooking oil	RWDC Industries Ltd., Athens, GA, USA	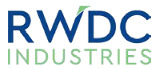	Solon^®^	4000 (expected to be expanded)	n.r.
	Rec. *C. necator* (“reprogrammed microorganism”) (salt lake isolate)	“Alternative carbon source, including crops and kitchen waste”, seawater	Bluepha Co. Ltd., Beijing, PR China	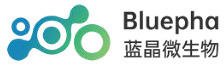	Bluepha PHA	1000	Readily degraded both in seawater and soil within 3–6 months
**Poly(4-hydroxybutyrate)** 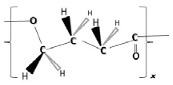	Rec. *Escherichia coli* (Enterobacterium)	4HB-related precursor	Tepha Medical Devices Inc., Lexington, MA, USA	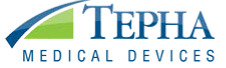	TephaFLEX	n.r.	FDA approved for biomedical use as implant material; the entire production process is ISO 13485 compliant

n.r.: not reported.

## Data Availability

Not applicable.
